# A 17-Year-Old Girl With Unilateral Headache and Double Vision

**DOI:** 10.1177/2324709619838309

**Published:** 2019-04-22

**Authors:** Larissa G. Rodriguez-Homs, Mark Goerlitz-Jessen, Samrat U. Das

**Affiliations:** 1 Duke University School of Medicine, Durham, NC, USA

**Keywords:** Tolosa-Hunt syndrome, pediatric neurology, pediatric neuro-ophthalmology

## Abstract

Tolosa-Hunt syndrome is characterized by a painful ophthalmoplegia secondary to a granulomatous inflammation in or adjacent to the cavernous sinus. Magnetic resonance imaging will show enhancement of the cavernous sinus and/or the orbital apex. Although this syndrome is extremely rare in children, it should be a diagnostic consideration in patients presenting with painful ophthalmoplegia with variable involvement of cranial nerves II to VI. The differential diagnosis for unilateral cavernous sinus lesion is broad, including vascular lesions (cavernous sinus thrombosis), inflammatory processes (sarcoidosis, autoimmune), neoplastic processes (schwannoma, lymphoma), as well as infectious etiologies. We describe a pediatric patient presenting with neurological symptoms from a unilateral cavernous sinus magnetic resonance imaging abnormality and the thorough diagnostic approach to arrive at the diagnosis of Tolosa-Hunt syndrome.

## Presentation

A 17-year-old girl with an unremarkable past medical history presents with 3 weeks of right-sided headache and horizontal binocular diplopia. She describes her pain as a “heaviness” located above the right eye accompanied with double vision. She was initially diagnosed with migraines and prescribed amitriptyline without improvement in symptoms. Thereafter, she went to an ophthalmologist and was diagnosed with a right cranial nerve VI palsy. A magnetic resonance imaging (MRI) of the brain with and without gadolinium was ordered and read as “normal.” She was then prescribed a 7-day prednisone taper and fioricet for migraine.

While on the prednisone, her headaches improved, but did not resolve. The diplopia persisted. On completion of the taper, her headaches worsened. Eleven days after the first study, a repeat MRI brain and orbits with/without gadolinium was obtained, revealing a possible cavernous sinus lesion. She denied head/eye trauma, nausea, vomiting, changes in gait or balance, sick contacts, fever, or recent illness.

At the time of initial admission, she was afebrile with normal vital signs. Physical examination revealed 20/20 vision, full color vision, full confrontation visual fields, and normal pupillary responses in both eyes. She demonstrated impaired abduction of the right eye with esoptropia. Remainder of the examination was unremarkable.

An initial workup revealed normal complete blood count, serum electrolytes, and an unremarkable chest X-ray. During the hospitalization, serum and cerebrospinal laboratory results were obtained.

## Discussion

**Figure 1.**
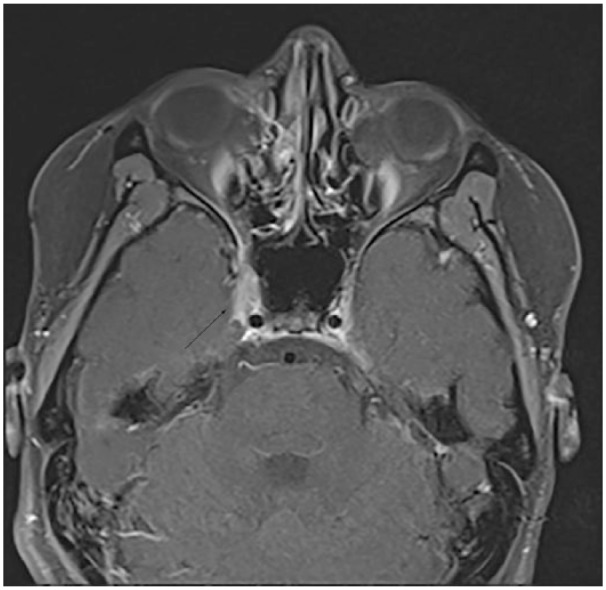
MRI of the orbits shows an asymmetric mass-like enhancement of right cavernous sinus region depicted by the black arrow.

The MRI brain with and without gadolinium showed a lesion of the right cavernous sinus affecting the right sixth cranial nerve (Figure 1). The differential diagnosis of right-sided headache, binocular diplopia, and cavernous sinus lesion on MRI includes cavernous sinus thrombus, inflammatory processes (sarcoidosis, IgG4 [immunoglobulin G4] disease), autoimmune disease (lupus, granulomatosis with polyangiitis), neoplasm (schwannoma, lymphoma, and metastatic disease), paraneoplastic syndrome, infectious (cryptococcal, mycoplasma, and herpes simplex virus), and idiopathic etiology.

An extensive workup was completed. Magnetic resonance angiogram and magnetic resonance venography were normal and ruled out cavernous sinus thrombosis. Anti-nuclear antibody, IgG subclasses, anti-neutrophil cytoplasmic antibody, anti-double stranded DNA, serum protein electrophoresis, cryptococcal antibodies, rapid plasma reagin, Lyme disease screen, mycoplasma antibodies, HIV antigen, and West Nile polymerase chain reaction were also normal. Cerebral spinal fluid (CSF) studies showed normal cell count, normal meningitis polymerase chain reaction panel, no growth on culture, normal α-fetoprotein level (to rule out hormone secreting germ cell tumor), no malignant cells, no oligoclonal bands, and normal angiotensin converting enzyme (ACE). Serum ACE and lysozyme were also measured to increase the sensitivity of sarcoid screening. Both were within normal limits.

Diagnostic finality based on serum and CSF testing was limited by the poor sensitivity of ACE and lysozyme in the diagnosis of sarcoid. Of note, computed tomography of the chest is more sensitive for sarcoidosis compared to chest X-ray. Although chest X-ray was completed, no computed tomography was performed.

Biopsy of the lesion in the right cavernous sinus was discussed but not performed as the potential complications of a biopsy outweighed the benefits. Because the patient’s neurological status remained stable throughout her admission, CSF cytology and serum protein electrophoresis returned negative, the diagnosis of an idiopathic inflammatory process, or Tolosa-Hunt syndrome (THS), was the concluded diagnosis rather than a neoplastic process such as lymphoma. Furthermore, our patient met the diagnostic criteria for THS as defined by the 2013 International Classification of Headache Disorders (Third Edition, ICHD-3 beta) diagnostic criteria.^1^ After multidisciplinary discussion with neurology, ophthalmology, and hematology/oncology, intravenous high-dose methylprednisolone, 1 g per day, was given for 3 days. Patient noted improvements in her headaches and diplopia. Patient’s abduction of the right eye had also slightly improved. She was discharged on a prolonged steroid taper with close ophthalmology follow-up.

## The Condition

THS is a granulomatous inflammatory process, adjacent to the cavernous sinus or within the superior orbital fissure and/or orbital apex characterized by infiltration of lymphocytes and plasma cells.^1^ What initiates the inflammatory reaction is still unclear.^2^ THS is considered a benign condition; however, there has to be clear exclusion of more malignant diseases. Knowing the differential diagnosis is important in order to rule out other potentially devastating causes of the presenting symptoms rather than ruling in the syndrome itself.^2^

## Clinical Features

THS is seen at any age without gender preference, and it is characterized by painful ophthalmoplegia described as a steady gnawing or boring pain.^3^ The syndrome is typically unilateral with bilateral symptoms occurring in 4% to 5% of the cases.^4^ Diplopia results from mononeuropathy or polyneuropathy. The third cranial nerve is most commonly involved (85% of cases) followed by the sixth cranial nerve (70%), the ophthalmic division of cranial nerve V (30%), and the fourth cranial nerve (29%).^4^ If the orbital apex is involved, patients may develop an optic neuropathy.^5^

## Diagnosis

The clinical diagnostic criteria for THS per ICHD-3^1^ guidelines include the following:

- An episode of unilateral headache with evidence of causation demonstrated by
  - Headache preceded paresis of the third, fourth, and/or sixth cranial nerves by ≤2 weeks, or developed with it
  - Headache is localized around the ipsilateral brow and eye
- Both of the following:
  - Granulomatous inflammation of the cavernous sinus, superior orbital fissure or orbit, demonstrated by MRI or biopsy
  - Paresis of one or more of the ipsilateral third, fourth, and/or sixth cranial nerves
- Exclusion of causes elicited by another ICHD-3 diagnosis

The combined clinical and radiologic criteria have high sensitivity for the diagnosis of THS (95% to 100%).^6^ THS tends to be an episodic illness spaced over many months lasting for a few weeks to months. This disorder is extremely rare and is reported with an incidence of one in a million individuals per year.^3^ This disease is especially rare in the pediatric population as the mean age of onset is reported as 38-41 ± 14-16 years.^5^

## Magnetic Resonance Imaging

Typical MRI findings include enhancement of the affected cavernous sinus and/or the orbital apex.^7^ These findings, however, are not unique to THS and may be found in other diseases such as lymphoma, sarcoidosis, and meningioma.^2^ The main utility of MRI with magnetic resonance venography of the brain is to exclude cavernous sinus thrombosis as well as intraorbital or intraparenchymal tumor.^2^

Biopsy of the lesion is the most definitive diagnosis; however, it is seldom necessary unless the patient does not respond to standard therapy and is clinically worsening.^8^ Follow-up MRI studies should be carried out to document resolution of the lesion with steroid therapy.^7^

## Treatment

Glucocorticoids are mainstay of treatment for THS and results in rapid resolution of pain.^9^ Motor signs due to cranial neuropathies may take a few weeks to resolve.^9^ There is no definite evidence that glucocorticoids help quicken the resolution of cranial nerve defects.^9^ In general, glucocorticoid regimens for THS treatment includes initial high-dose glucocorticoids, up to 1 g per day, for 2 to 4 weeks followed by a gradual taper for a period of 4 to 6 weeks.^9^ The eye examination dictates the tapering. Close clinical follow-up with repeated MRI every 1 to 2 months is recommended to ensure response to therapy and to ensure no other etiology develops.^10^

Although THS is often a benign condition, it has the potential to cause blindness due to optic nerve involvement.^5^ Therefore, pediatricians must be able to recognize the symptoms and clinical features of THS. This will allow for immediate treatment to preserve vision and ocular motility function as well as to prevent sequelae at a later stage.^5^

## Lessons for the Clinician

1. THS is a painful ophthalmoplegia, which is extremely rare in the pediatric population.
2. A systematic approach to the diagnosis of painful ophthalmoplegia is required to arrive at this diagnosis and is presented above.
3. The condition can be diagnosed based on clinical presentation and neuroradiological findings.
